# Efficacy of blended digital and face-to-face psychotherapy compared to enhanced psychotherapy for patients with Somatic Symptom Disorder (iSOMA+): study protocol for a multicenter randomized controlled pragmatic trial

**DOI:** 10.1186/s13063-026-09886-y

**Published:** 2026-07-10

**Authors:** Freda Kristine Jutzi, Michael Witthöft, Harald Baumeister, Mira Denninger, Winfried Rief, Gaby Bleichhardt, Josef Bailer, Peter Kirsch, Maja Halli-Erkic, Daniela Mier, Mara Mankin, Julia A. Glombiewski, Rabea Vogt, Jens Heider, Frank Jacobi, Alexandra Martin, Barbara Schulte-Holthausen, Kai Kronfeld, Stanislav Gorbulev, Christian Ruckes, Rüdiger Pryss, Robin Kraft, Severin Hennemann

**Affiliations:** 1 Department of Clinical Psychology, Psychotherapy and Experimental Psychopathology, Johannes Gutenberg-University Mainz, Mainz, Germany; 2 Department of Clinical Psychology and Psychotherapy, Ruhr University Bochum, Bochum, Germany; 3 Department of Clinical Psychology and Psychotherapy, Institute of Psychology and Education, Ulm University, Ulm, Germany; 4 Department of Clinical Psychology and Psychotherapy, University of Marburg, Marburg, Germany; 5 Department of Clinical Psychology, Medical Faculty Mannheim, Central Institute of Mental Health/University of Heidelberg, Mannheim, Germany; 6 Department of Clinical Psychology and Psychotherapy, University of Konstanz, Konstanz, Germany; 7 Department of Clinical Psychology and Psychotherapy, RPTU University Kaiserslautern-Landau, Landau, Germany; 8 Department of Clinical Psychology and Psychotherapy, Psychologische Hochschule Berlin, Berlin, Germany; 9 Department of Clinical Psychology and Psychotherapy, University of Wuppertal, Wuppertal, Germany; 10 Interdisciplinary Center Clinical Trials (IZKS), University Medical Center Mainz, Mainz, Germany; 11 Institute of Clinical Epidemiology and Biometry, University of Würzburg, Würzburg, Germany; 12 Institute of Medical Data Science, University Hospital of Würzburg, Würzburg, Germany

**Keywords:** Persistent somatic symptoms, Somatic Symptom Disorder, Blended psychotherapy, Internet intervention, Randomized controlled trial

## Abstract

**Background:**

Persistent somatic symptoms (PSS) that occur in various somatic, functional, or mental health conditions can lead to considerable psychological distress and functional impairment. As such, they are a key characteristic of the Somatic Symptom Disorder (SSD) in DSM-5. While psychological treatments such as cognitive behavioral approaches can address these symptoms, their clinical efficacy in reducing symptom burden in previous trials remains limited. Blended psychotherapy, i.e., combining face-to-face psychotherapy with digital elements, is a promising new approach to efficiently enhance psychotherapeutic effects. This study therefore aims to evaluate the efficacy, mechanisms, and safety of blended psychotherapy compared to enhanced standard psychotherapy for individuals with SSD in outpatient psychotherapy.

**Methods:**

A two-armed, multicenter randomized controlled pragmatic trial will be conducted, and *N* = 250 adults with SSD will be randomized to either blended psychotherapy (20 individual sessions of cognitive behavioral therapy (CBT) + accompanying digital intervention; iSOMA+) or enhanced CBT (20 sessions of CBT + self-help booklet; CBT+). Participants are recruited at eight German university outpatient psychotherapy clinics. Assessments will be conducted at patient study inclusion (pre-treatment), during treatment, post-treatment, and 6 months follow-up. The primary outcome is the reduction in somatic symptom severity using the Screening for Somatoform Disorders (SOMS-7R) from baseline to post-treatment. Secondary outcomes include changes in symptom-related distress, coping, self-efficacy, as well as depression, anxiety, health anxiety, disability, quality of life, interpersonal relationship experiences and patient safety. Additionally, several potential moderators and mediators, including patient and intervention characteristics, will be examined.

**Discussion:**

This trial investigates the potential of blended CBT for improving treatment outcomes in patients with SSD and will provide evidence on the effects of active vs. passive self-help as treatment augmentation under pragmatic care conditions. By identifying prescriptive factors of treatment response, the study will support personalized care and contribute to more accessible and efficacious treatment options for patients with PSS.

**Trial registration:**

German Clinical Trials Register (DRKS) DRKS00035250. Registered on 13.06.2025, https://drks.de/search/de/trial/DRKS00035250.

## Administrative information

Note: the numbers in curly brackets in this protocol refer to SPIRIT checklist item numbers. The order of the items has been modified to group similar items (see http://www.equator-network.org/reporting-guidelines/spirit-2013-statement-defining-standard-protocol-items-for-clinical-trials/).

**Table:** 

Title {1}	Efficacy of blended digital and face-to-face psychotherapy compared to enhanced psychotherapy for patients with Somatic Symptom Disorder (iSOMA+): Study protocol for a multicenter randomized controlled pragmatic trial
Trial registration {2a and 2b}	The trial is registered at the German clinical trials registry DRKS: DRKS00035250 (https://drks.de/search/de/trial/DRKS00035250). The trial was registered on 13.06.2025.
Protocol version {3}	2026-06-18. Version 2.0.
Funding {4}	This study is funded by the German Research Foundation (DFG, Clinical Studies, Project Number 543351897).
Author details {5a}	Freda Kristine Jutzi, Department of Clinical Psychology, Psychotherapy and Experimental Psychopathology, Johannes Gutenberg-University Mainz, Mainz Germany Michael Witthöft, Department of Clinical Psychology and Psychotherapy, Ruhr University Bochum, Bochum, Germany Harald Baumeister, Department of Clinical Psychology and Psychotherapy, Institute of Psychology and Education, Ulm University, Ulm, Germany. Mira Denninger, Department of Clinical Psychology and Psychotherapy, Ulm University, Ulm, Germany Winfried Rief, Department of Clinical Psychology and Psychotherapy, University of Marburg, Marburg, Germany Gaby Bleichhardt, Department of Clinical Psychology and Psychotherapy, University of Marburg, Marburg, Germany Josef Bailer, Department of Clinical Psychology, Central Institute of Mental Health, Medical Faculty Mannheim/University of Heidelberg, Mannheim, Germany Peter Kirsch, Department of Clinical Psychology, Central Institute of Mental Health, Medical Faculty Mannheim/University of Heidelberg, Mannheim, Germany Maja Halli-Erkic, Department of Clinical Psychology, Central Institute of Mental Health, Medical Faculty Mannheim/University of Heidelberg, Mannheim, Germany. Daniela Mier, Department of Clinical Psychology and Psychotherapy, University of Konstanz, Konstanz, Germany Mara Mankin, Department of Clinical Psychology and Psychotherapy, University of Konstanz, Konstanz, Germany Julia A. Glombiewski, Department of Clinical Psychology and Psychotherapy, RPTU University Kaiserslautern—Landau, Landau, Germany Rabea Vogt, Department of Clinical Psychology and Psychotherapy, RPTU University Kaiserslautern—Landau, Landau, Germany Jens Heider, Department of Clinical Psychology and Psychotherapy, RPTU University Kaiserslautern – Landau, Landau, Germany Frank Jacobi, Department of Clinical Psychology and Psychotherapy, Psychologische Hochschule Berlin, Germany Alexandra Martin, Department of Clinical Psychology and Psychotherapy, University of Wuppertal, Wuppertal, Germany Barbara Schulte-Holthausen, Department of Clinical Psychology and Psychotherapy, University of Wuppertal, Wuppertal, Germany Kai Kronfeld, Interdisciplinary Center Clinical Trials (IZKS), University Medical Center Mainz, Germany Stanislav Gorbulev, Interdisciplinary Center Clinical Trials (IZKS), University Medical Center Mainz, Germany Christian Ruckes, Interdisciplinary Center Clinical Trials (IZKS), University Medical Center Mainz, Germany Rüdiger Pryss, Institute of Clinical Epidemiology and Biometry, University of Würzburg, Würzburg, Germany, and Institute of Medical Data Science, University Hospital of Würzburg, Würzburg, Germany Robin Kraft, Institute of Clinical Epidemiology and Biometry, University of Würzburg, Würzburg, Germany, and Institute of Medical Data Science, University Hospital of Würzburg, Würzburg, Germany Severin Hennemann, Department of Clinical Psychology, Psychotherapy and Experimental Psychopathology, Johannes Gutenberg-University Mainz, Germany
Name and contact information for the trial sponsor {5b}	German Research Foundation (DFG, GZ HE 8867/3-1)
Role of sponsor {5c}	No sponsor has a part in the study design, data collection, management, analysis and interpretation, writing of the report, or decision to submit the report.

## Introduction

### Background and rationale {6a}

Persistent somatic symptoms (PSS), that occur on most days over several months, including but not limited to pain, fatigue, bowel dysfunctions, palpitations, or others, are widespread in the general population [1, 2], and even more prevalent in medical settings, where rates of up to 49% have been reported [3]. PSS may develop following medical conditions (e.g., infections, injuries) or procedures (e.g., surgeries), after stressful life events, or de novo, with the onset and even more their persistence assumed to result from an interplay of various biopsychosocial risk factors [4]. PSS can be associated with significant impairment [5] and psychological distress [6, 7], as mirrored in increased disability-adjusted life years and health care costs [8, 9]. PSS can be present in various somatic, functional, and psychological conditions and as such are a central feature of various disease classifications, including Somatic Symptom Disorder (SSD) in DSM-5. SSD is characterized one or more distressing and/or impairing somatic symptoms accompanied by excessive and dysfunctional cognitive (e.g., worrying), affective (e.g., health anxiety), or behavioral reactions (e.g., frequent medical care utlization). While prevalence rates are lower than for the much broader concept of PSS, SSD, and former, related diagnostic concepts can be considered one of the most common and burdensome mental disorders in most western countries [10], affecting 5–7% of the general population [11] and up to 78% in clinical populations [6]. Comorbidity with other mental disorders is common [11–13], and suicidality is frequently associated [14, 15]. Treatment guidelines recommend a primary care-centered biopsychosocial approach that includes addressing the underlying pathophysiology, person-centered communication techniques, and symptomatic pharmacological relief [16]. In more complex cases, for example, with debilitating somatic symptoms and/or severe psychological comorbidity, targeted psychological and pharmacological interventions are indicated [17, 18]. Among these, the best evidence exists for cognitive behavioral therapy (CBT) in reducing symptom severity, distress, and functional impairment [19].

However, the efficacy of psychotherapeutic treatment options remains suboptimal, with only small to moderate effects compared to control groups according to meta-analyses of previous RCTs [18, 20]. Moreover, access to adequate care remains challenging [21], with patients often perceived as “difficult to treat” and many affected individuals remain psychologically untreated [21, 22]. To address these challenges, innovative treatment approaches are required. Blended psychotherapy, which combines face-to-face therapy with digital therapeutic elements, represents a promising new approach that might enhance the efficacy (i.e., boosting treatment effects) or efficiency (i.e., reallocating therapeutic resources) of psychotherapy [23]. Blended interventions may be used sequentially (e.g., stepped-care models in pre- or post-treatment phase) or integrated into ongoing therapy [23, 24]. Compared to stand-alone digital programs, which have proven to be effective but limited in uptake and acceptance [25–28], blended formats offer greater therapeutic range and higher acceptance by both patients and therapists [26, 29–31]. While blended psychotherapy is a rather new field in mental health care, one of the first systematic reviews, particularly focusing on depression, anxiety, and substance use disorders, provides evidence for its effectiveness, showing reduced drop-out rates and decreased therapist time requirements [23]. Integrated blended psychotherapy shows positive effects for depression, but mixed or non-significant results for anxiety disorders [32]. Furthermore, meta-analytic findings support the augmentation of treatment effects of face-to-face therapy through the additional use of mobile technologies across various disorders, compared to standard treatment [33]. For example, Berger et al. [34] found that a combination of outpatient psychotherapy with an additional unguided online intervention was more effective than face-to-face therapy alone in patients with depression, yielding a medium-sized effect. In the context of chronic somatic conditions, previous combined care approaches have primarily focused on supplementing primary medical care with brief digital self-management programs, yielding mixed evidence regarding their effectiveness [35, 36]. To date, no systematic evaluation of blended psychotherapy for PSS or SSD exists. Given the limited efficacy of previous short-term psychological interventions [37] and the positive dose-response relation for treatment success [38], an integrated blended psychotherapy may offer a resource-efficient and scalable path to improving outcomes in secondary (specialized) care for SSD beyond the primary care level.

### Objectives {7}

The primary objective of this trial is to investigate the efficacy, that is to show that an integrated form of blended psychotherapy (iSOMA+) reduces somatic symptom severity significantly stronger than CBT enhanced with bibliotherapy (CBT+) from baseline to post-treatment.

Furthermore, we expect that iSOMA+, compared to CBT+, will lead to significantly greater reductions in secondary outcomes, including somatic symptom severity, adverse consequences of somatic symptoms, depression, anxiety, health anxiety and disability as well as significantly greater improvements in self-efficacy, somatic symptom coping, quality of life and experience in personal social systems from baseline to post-treatment and from baseline to the 6-month follow-up.

Thirdly, we expect that iSOMA+, compared to CBT+, will lead to a significantly higher rate of meaningful clinical change (response, remission) in somatic symptom severity.

Fourthly, we will investigate mechanisms of action, since previous trials have identified that motivational (e.g., self-efficacy, treatment agency) and functional (e.g., pain-related disability) characteristics are positively associated with treatment outcomes and may act as mediators of therapeutic change [39–41]. However, their role in blended psychotherapy is unclear. Therefore, we test whether an increase in self-efficacy, treatment agency, and a reduction in functional impairment will mediate the treatment effect of iSOMA+ compared with CBT+.

Exploratively, we will investigate to what extent (a) demographic characteristics of patients (e.g., gender), (b) clinical characteristics (e.g., baseline severity of somatic symptom distress, comorbidity), and (c) treatment-related factors (e.g., concurrent treatments, treatment expectations) moderate the treatment effect between the two treatment conditions (iSOMA+ vs. CBT+).

Furthermore, we will examine whether iSOMA+ differs from CBT+ with respect to changes in health care utilization from baseline to the end of treatment and from baseline to the 6-month follow-up. Treatment satisfaction and implementability will be tested. Finally, patient safety will be investigated, and no significant differences are expected in the type or frequency of serious adverse events (SAEs) and other side-effects between iSOMA+ and CBT+.

### Trial design {8}

**Fig. 1:**
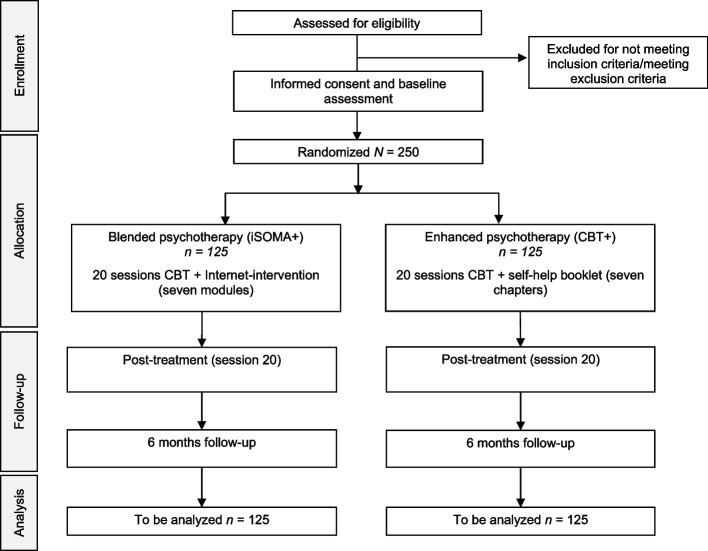
Study flow chart

This is a prospective, multicenter, two-armed, active-controlled, parallel group, randomized controlled superiority trial that will be conducted at eight university outpatient psychotherapy centers in Germany. A total of 250 adult patients with SSD will be included, 125 patients per study arm. Participants will be randomly allocated to the two conditions. Both study groups receive 20 sessions of CBT. Additionally, the intervention group receives the iSOMA internet intervention, while the active control group receives a self-help booklet. Primary and secondary outcomes will be measured before randomization (baseline), during the course of treatment, after completion of the face-to-face treatment (post-assessment), and 6 months after end of treatment (follow-up) (see Fig. 1).

## Methods: participants, interventions, and outcomes

This protocol manuscript follows the SPIRIT reporting guidelines and the SPIRIT checklist is attached [42].

### Study setting {9}

Eight German university outpatient clinics for psychotherapy will serve as study centers, based at the Johannes Gutenberg University Mainz, Central Institute of Mental Health, Medical Faculty Mannheim/University of Heidelberg, Philipps University Marburg, RPTU University Kaiserslautern – Landau, Ulm University, Bergische University Wuppertal, University of Konstanz and Psychologische Hochschule Berlin. All centers are involved in patient recruitment and the implementation of study therapies. In all centers, treatment will be offered in an outpatient setting.

### Eligibility criteria {10}

Adults (≥ 18 age) diagnosed with SSD (according to DSM-5) who also exhibit a sufficient level of symptom severity, as indicated by a score ≥ 5 in the Patient Health Questionnaire, somatic symptom version (PHQ-15 [43]), and disability, as indicated by a score ≥ 4 in the (modified) Pain Disability Index (PDI [44]), and who have access to the internet and an internet-capable device, will be eligible for inclusion. The selected PHQ-15 cut-off has been shown to be a suitable marker for identifying individuals at high risk for a diagnosis of SSD in a representative population sample [45]. Similarly, the PDI cut-off has proven to be sensitive to relevant impairments in functional status due to PSS in a population-based survey [44]. Prior to their inclusion in the study, written informed consent will be obtained from all participants (inclusion criteria). Participants who meet at least one of the following exclusion criteria will be unable to enroll in the trial (exclusion criteria): (a) Acute, unstable, or severe physical condition that prevents safe trial participation, (b) inadequate command of the German language (reading and/or speaking) (c) severe substance use disorder (according to DSM-5), (d) acute suicidality or (e) acute psychotic states, (f) parallel psychotherapy or (g) participation in another intervention study. The study therapists will be licensed CBT therapists or clinical psychologists currently undergoing advanced post-graduate CBT training under supervision. Prior to treating study patients, all therapists will complete study-specific training provided by the principal investigator (PI) about SSD, the treatment conditions, and the study procedures. For ethical considerations, the study does not include a no-treatment control group.

### Who will take informed consent? {26a}

Individuals who are potentially eligible and interested after pre-screening are invited to participate in an interview with an independent, trained clinical psychologist from the study team. During this interview, the participants are informed in detail about the study and will be given time and opportunity for questions and concerns. The information is provided in both verbal and written formats. The following aspects are explained: (a) the objective of the study and its scientific relevance, (b) the procedure and duration of participation in the study, (c) potential risks or side effects, (d) possible benefits, (e) collection and processing of study data (including data security), and (f) the right to terminate participation in the study at any time without giving reasons and without negative consequences. Eligible participants who are willing to participate will then sign informed consent. The informed consent materials and forms are available from the corresponding author on request.

### Additional consent provisions for collection and use of participant data and biological specimens {26b}

Not applicable, the trial does not involve collecting biological specimens.

## Interventions

### Explanation for the choice of comparators {6b}

The intervention group (blended psychotherapy) will be compared to an active psychotherapy control group. CBT is considered a standard, theory- and evidence-based psychological treatment in outpatient or multidisciplinary inpatient settings [16, 19, 20, 46, 47]. In the control group, CBT will be combined with self-help bibliotherapy. Previous studies have demonstrated positive effects of bibliotherapy, but these have predominantly focused on functional somatic syndromes [48], indicating a research gap for the broader diagnostic spectrum of SSD. For instance, Hedman et al. [49] found comparable effects between bibliotherapy and internet-based CBT in reducing health anxiety among patients with SSD and illness anxiety disorder. However, this study exclusively targeted health anxiety and bibliotherapy was implemented as stand-alone treatment, without integration into face-to-face therapy. In the current study, the structure, content and dosage of the bibliotherapy (consisting of 7 chapters) will be practically matched to the supplementary internet intervention (7 consecutive modules), allowing differentiation between effects due to delivery format and those due to treatment dosage. In case of ethical requirements (e.g., crisis intervention), participants in both study groups may receive up to four additional “joker sessions” on top of the manualized sessions. Likewise, continuation of treatment beyond the study therapy may be permitted based on clinical judgment for ethical reasons and will be monitored accordingly. Nonetheless, the superiority of blended psychotherapy is assumed, given the multimedial and interactive design as well as further persuasive, that is, engagement-enhancing, features of iSOMA (i.e., automatic reminders, progress bar, unlocking achievements), compared to passive bibliotherapy [50].

### Intervention description {11a}

In both conditions, patients receive health insurance-covered outpatient individual psychotherapy, based on an established and efficacious CBT manual for PSS and somatoform disorders [51–53]. Therapy sessions take place on an approximately weekly basis, and overall treatment duration is thus estimated with a minimum of 6 months (including possible intervals). The therapeutic concept targets characteristic perpetuating cycles between cognitive, behavioral, and physiological responses that maintain symptoms, distress, and disability according to cognitive-behavioral and somatosensory models of PSS [54, 55]. An overarching goal of the treatment is to enhance patients’ motivation to explore further biopsychosocial explanations for their bodily symptoms and to identify self-efficacious strategies for reducing and managing symptoms and functional disability. Cognitive modifications include reducing selective attention to bodily processes by fostering greater flexibility in perception and experience, broadening symptom attributions and developing constructive evaluation patterns in response to symptom experiences (e.g., changing expectations or self-instructions), as well as promoting a realistic, dimensional understanding of illness and health. On a psychophysiological level, the treatment seeks to lower autonomic arousal through regenerative stress management techniques, particularly by relaxation training. Behaviorally, the treatment aims to reduce illness behaviors by developing physical resilience and balanced activity level, helping patients to replace maladaptive patterns such as avoidance or protective inactivity, and excessive safety behaviors (e.g., medical reinsurance) [52, 53]. An overview of the treatment protocol is given in Table 1.

**Table 1:** Overview of treatment sessions

Regular psychotherapy	Treatment content	iSOMA online modules/self-help booklet chapters
Session 1–2	Symptom monitoring, goal setting	My compass
Session 3–4	Education on stress reaction (e.g., autonomous nervous system, symptom influence), relaxation exercises	Stress and the body
Session 5–7	Attention modification, behavioral activation	Shifting focus
Session 8–12	Cognitive restructuring	Changing perspectives
Session 13–16	Reduction of illness behavior (e.g., reinsurance-, safety, avoidance behavior), graded physical exercise	Finding strength
Session 17–19	Stress management, education on transactional stress model, stress-coping, communication skills	Solving problems and strengthening relationships
Session 20	Summary and self-management	Fit for the future

*Intervention group (blended psychotherapy):* In addition to the face-to-face treatment protocol, participants receive the accompanying internet intervention (iSOMA), consisting of seven modules and a technical introduction. Thus, iSOMA mirrors a chronological, mostly standardized self-help intervention. The content and scheduling of the iSOMA modules are tailored to the face-to-face session protocol and show a high degree of content overlap, reinforcing treatment transfer and multimodal learning (see Table 1). Each of the iSOMA modules includes standardized elements of psychoeducation, exercises, behavioral experiments, and assignments, which are presented in a multimedia format via text, audio, or video in an interactive form (e.g., free text fields for personal experience, preset feedback for multiple-choice questions, quizzes). Fictional patient examples (e.g., gastrointestinal, pain-related symptomatology, avoidant or endurance type) are used to illustrate typical problems and therapeutic principles. In addition, persuasive design features such as automatic reminders, tailored progress tracking and aspects of gamification (e.g., achievable skills) are incorporated. The modules can serve both as primers and enhancements of face-to-face sessions and are activated consecutively by therapists within the corresponding treatment sections, thus following a semi-flexible integration mode, which for example was recently studied in outpatient psychotherapy for depression and anxiety as part of the PSYCHOnlineTHERAPIE trial [56]. The ratio of treatment sessions to digital modules is approximately 3:1 (see Table 1). Patients work through the content between sessions using their own devices (e.g., PC, laptop, tablet, smartphone), while having the option to save their progress intermittently. Therapists can monitor the patient’s progress in the online modules and are encouraged to integrate content such as digital worksheets and exercises into treatment sessions. While initial contact via the platform is technically possible for therapists, it is neither intended nor permitted as part of the study protocol. According to the findings of preceding studies [57], the estimated processing time for each online-module ranges from approximately 45 to 60 min. iSOMA builds on a previously evaluated intervention form, tested as a guided, cognitive-behavioral, modular online program for PSS (non-blended). In a randomized controlled trial with 156 emerging adults (mean age 24 years, 83% female), iSOMA led to significantly greater reductions in somatic symptom severity (PHQ-15: *d* = 0.70) and SSD-related distress (SSD-12: *d* = 0.65) compared to a waitlist control group [57]. In a subsample with clinically relevant levels of self-reported somatic symptom distress (*n* = 83), similar effects than for the total sample were observed (PHQ-15: *d* = 0.68; SSD-12: *d* = 0.72). The implementation of the internet intervention utilizes the university-based, open-source eHealth platform “eSano”, which was developed by a member of the trial steering team (HB) and IT-expert (RP) [58, 59]. The platform was developed with consideration of the regulatory frameworks defined by the German Medical Devices Act and the European Union Medical Device Regulation. The software development and validation process was guided by internationally recognized standards, including IEC 62304 (classified as safety class B), GAMP5 (category 4), the FDA's General Principles of Software Validation, and the Pharmaceutical Inspection Co-operation Scheme guideline 11-3 [56].

**Fig. 2:**
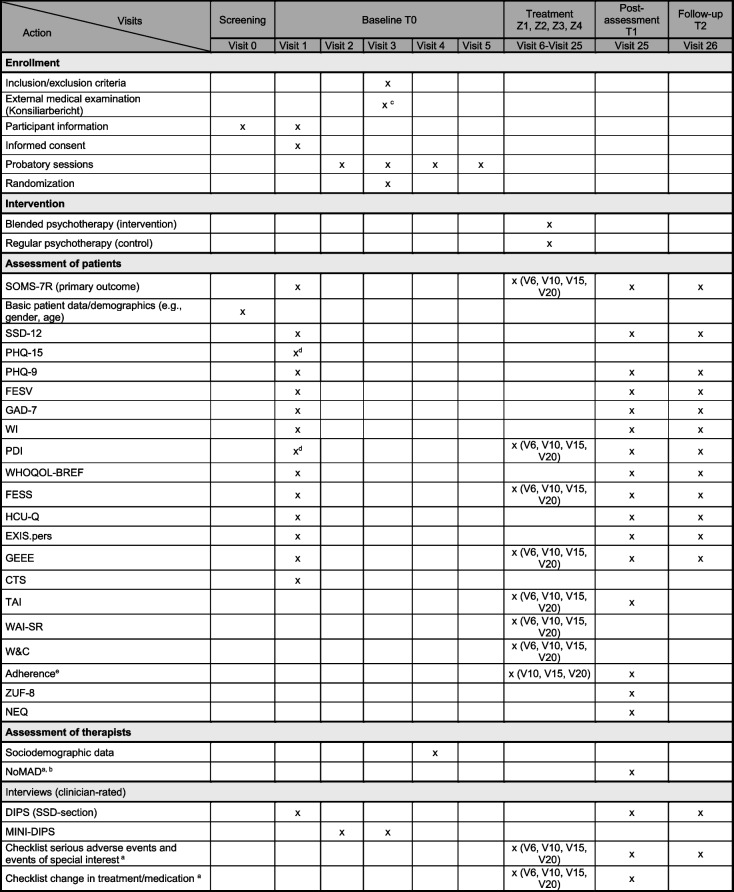
Visit plan. DIPS, Diagnostic Interview for Psychological Disorders [61]; Mini-DIPS, short form of the Diagnostic Interview for Psychological Disorders [62]; CTS, Childhood Trauma Screener [63]; EXIS.pers, Experience in Personal Social Systems Questionnaire [64]; TAI, Therapeutic Agency, therapeutic-processing subscale [65]; NEQ, Negative Effects Questionnaire – 20 Item Version [66]; NoMAD, Normalization Measure Development questionnaire [67]; GEEE, Generic rating scale for previous treatment experiences, treatment expectations, and treatment effects [68]; W&C, Warmth and Competence Scale [69]; SOMS-7R, Screening for Somatoform Disorders – revision [70, 71]; WAI-SR, Working Alliance Inventory [72]; SSD-12, Somatic Symptom Disorder – B Criteria Scale [73]; PHQ-9, Patient Health Questionnaire – depression scale [74]; FESV, Pain Coping Questionnaire – coping subscales [75]; GAD-7, Generalized Anxiety Disorder Questionnaire [76]; WI, Whiteley Index [77]; WHOQOL-BREF, World Health Organization Quality of Life questionnaire [78]; PDI, Pain Disability Index [44]; FESS, Pain Self-Efficacy Questionnaire [79]; HCU-Q, Health Care Utilization Questionnaire [80]; ZUF-8, Client Satisfaction Questionnaire [81]; ^a^filled out by therapists; ^b^only in intervention condition; ^c^this external examination should be conducted up to visit 2 and visit 3 at the latest; ^d^assessed as part of the pre-screening; ^e^In both study arms, adherence and intervention usage will be continuously assessed throughout the treatment

*Control group:* In addition to the face-to-face treatment protocol, patients receive a self-help booklet, corresponding to the structure and content of the online modules (see Table 1). It contains information material, exercises, and worksheets intended for self-help. The booklet is handed out by therapists at treatment initiation (visit 4, see Fig. 2).

### Criteria for discontinuing or modifying allocated interventions {11b}

In the event that clinical circumstances arise during treatment that render continuation of the allocated intervention inappropriate, unsafe, or a strictly manualized approach ethically untenable, the therapist and supervisor (see subsequent section) may jointly decide to discontinue or modify the intervention and continue treatment within routine care. Such circumstances may include acute suicidality, acute psychotic symptoms, severe psychological or physical deterioration, withdrawal of consent by the participant, parallel psychotherapeutic treatment, enrolment in another clinical trial or research project that is not scientifically or medically compatible with the present study, or persistent non-compliance with treatment or study procedures that precludes protocol-compliant participation. Participants may discontinue the intervention at any time without providing a reason and without any disadvantages. Participants are invited to post-treatment assessment and the follow-up assessment even in case of intervention discontinuation.

### Strategies to improve adherence to interventions {11c}

Both interventions are manualized, and therapists will be trained in both treatment forms. All treatments are video- (or audio-)taped and - in case of trainee therapists - continuously supervised (approximately every fourth session) by licensed supervisors experienced in the CBT rationale [51]. Licenced psychotherapists will schedule regular intervision groups. Adherence and engagement with the online modules will be automatically assessed within the platform. Therapists can monitor their patients’ platform usage (e.g., lecture/diary completion) and address potential issues in personal sessions. In the control group, patients will report their use of the self-help booklet regularly during the treatment process. Treatment integrity will be video- (or audio-)registered and rated after completion of the treatments using the method of assessing treatment delivery (MATD) in clinical trials [60]. Although therapists deliver both treatment forms, treatment contamination can practically be ruled out since therapists cannot alter the add-on (iSOMA vs. self-help booklet). Patients in the intervention group receive automatic e-mail notifications if a new module has been assigned to them or if they have not completed a module within ten days.

### Relevant concomitant care permitted or prohibited during the trial {11d}

If suicidality requires acute treatment (e.g., referral to inpatient treatment), patients will be excluded from this trial, and these events will be monitored. Additional psychotherapy is not allowed. In principle, all other forms of treatment (except parallel psychotherapy) are permitted, including the use of medication, as this reflects the reality of clinical practice. Accompanying treatments are recorded and changes are regularly monitored (see Fig. 2).

### Provisions for post-trial care {30}

There is no ancillary post-trial care or compensation specifically related to study participation beyond routine care. Participants are covered by participant insurance and travel insurance for any study-related harms. Participants receive guideline-approved outpatient psychotherapy within the German statutory health care system throughout the study period. If clinically significant psychological distress or ongoing treatment needs remain after completion of the intervention (session 20), continued treatment within routine care will be recommended. Depending on individual clinical needs, participants may continue psychotherapy or be referred to access additional appropriate psychotherapeutic, psychiatric, psychosocial, or counselling services. Decisions regarding further care will be made by the treating psychotherapist and supervisor in accordance with clinical needs and standard practice.

### Outcomes {12}

The selection of outcome measures was based on the guidelines for clinical trials in patients with PSS by the European Research Network to Improve Diagnosis, Treatment and Health Care for Patients with Persistent Somatic Symptoms (EURONET-SOMA) [82]. Of main interest is the absolute change in scores in patient reported questionnaires from baseline (t0) to end of treatment (t1), the latter constituting the primary endpoint. The *primary outcome* will be self-reported somatic symptom severity, assessed using the revised version of the Screening for Somatoform Disorders (SOMS-7R [70, 71]) at baseline, during treatment (face-to-face sessions 1, 5, 10, and 15), post-treatment, and up to 6-month follow-up. Somatic symptom severity represents one of the core outcome domains in both research and clinical practice and is of high relevance to patients. It is considered as a continuous score and of primary interest is the absolute change from baseline (t0) to end of treatment (t1). The treatment differences will be displayed by a linear contrast for each study visit from the model with their 95% confidence intervals. The primary analysis population is the intention-to-treat (ITT) population consisting of all patients randomized. Long-term maintenance of the primary outcome, somatic symptom severity, will be evaluated using SOMS-7R scores at the 6-month follow-up. Additionally, we will qualify response and remission by change scores of the primary outcome (SOMS-7R) and remission by change in diagnostic status from baseline up to follow-up as categorical change parameters.

*Secondary outcomes* will be assessed at baseline, post-treatment and at the 6-month follow-up and will include the following constructs and questionnaires: adverse psychobehavioral consequences of somatic symptoms (Somatic Symptom Disorder – B Criteria Scale, SSD-12 [73]), symptom coping (Pain Coping Questionnaire, FESV, coping subscales [75]), health related self-efficacy (Pain Self-Efficacy Questionnaire, FESS [79]), depression (Patient Health Questionnaire – depression scale, PHQ-9 [74]), anxiety (Generalized Anxiety Disorder, GAD-7 [76]), health anxiety (Whiteley Index, WI [77]), disability (Pain Disability Index, PDI [44]), quality of life (World Health Organization Quality of Life questionnaire, WHOQOL-BREF [78]), and experience in personal social systems (Experience in Personal Social Systems Questionnaire, EXIS.pers [64]). *Mediators* of treatment effects will include self-efficacy (assessed via FESS), treatment agency (assessed via the therapy-oriented processing subscale of the Therapeutic Agency Inventory, TAI [39, 65]), and disability (assessed via PDI). Mediators will be assessed at baseline, during treatment (sessions 1, 5, 10, 15), post-treatment, and at follow-up. *Potential moderators* of the treatment-related change processes will be explored. These will be assessed at baseline and will include (a) demographic variables, (b) clinical characteristics (e.g., comorbidity, baseline symptom severity, traumatic experience via the Childhood Trauma Screener (CTS [63])), and (c) treatment characteristics (e.g., parallel treatments, type of referral, preferences, treatment expectations assessed via the Generic rating scale for previous treatment experiences, treatment expectations, and treatment effects (GEEE [68])). Health care utilization (Health care Utilization Questionnaire, HCU [80]) will be assessed at baseline, post-treatment and at follow-up. In addition, therapist behavior and therapeutic alliance will be assessed during treatment (sessions 1, 5, 10, and 15) using the Warmth & Competence Scale (W&C [69]) and the Working-Alliance Inventory – short revised (WAI-SR [72]). General treatment satisfaction will be assessed post-treatment using the Client Satisfaction Questionnaire (ZUF-8 [81]), and module-specific satisfaction and perceived effectiveness of iSOMA will be assessed using self-constructed items. The extent to which iSOMA is a normal part of the daily working routine of therapists will be assessed post-treatment with the Normalization Measure Development questionnaire (NoMAD [67]). In both study conditions, adherence and duration of intervention use will be continuously monitored throughout the treatment period using self-constructed items. Self-rated negative treatment effects will be assessed post-treatment by the Negative Effects Questionnaire (NEQ [66]). Psychotherapists will provide sociodemographic data and indicate treatment preference at baseline and monitor SAEs and events of special interest (ESIs) and protocol deviations throughout treatment (sessions 1, 5, 10 and 15), post-treatment and at follow-up (administered by study personnel).

### Participant timeline {13}

Individuals expressing interest will be subjected to a telephone (or in-person) interview to determine preliminary inclusion and exclusion criteria (pre-screening), accompanied by filling out questionnaires for screening purpose (basic patient data, PHQ-15, PDI). Preliminary eligible individuals will then be invited to a personal informational interview. During the course of the interview, the participants will be provided with comprehensive information and written consent will be obtained. The diagnosis of SSD will be confirmed based on the Diagnostic Interview for Psychological Disorders (DIPS; [61]), which will be administered by independent, experienced therapists. If eligibility criteria are met and the patient agrees to participate by signing the informed consent, they will be asked to complete the baseline survey (t0). Afterwards, participants will be assigned to their study therapist and the regular, health insurance-covered therapy regimen will continue, starting with up to four probatory sessions. In the initial two probatory sessions, which are mandatory preliminary appointments in the German health care system to assess diagnosis, treatment indication, and therapeutic fit, the study therapists will continue the diagnostic process using the short form of the diagnostic interview (Mini-DIPS; [62]) to assess potential exclusion criteria (SSD not main diagnosis) or further comorbid diagnoses. Patients will be referred for routine care medical check-up (“Konsiliarbericht”) to identify medical red flags that prevent safe trial participation. In cases of uncertainty, a medical supervisor will be available for consultation regarding the clarification of potential red flags. If all inclusion criteria are fulfilled, participants will be randomly assigned to one of the two study arms automatically within the electronic database (randomization, allocation). In the third probatory session, participants in the intervention group will be granted access to iSOMA, while those in the control group will be provided with the self-help booklet. Patients will then receive 20 sessions of blended psychotherapy or enhanced psychotherapy, respectively, in accordance with the session protocol depicted in Table 1. At therapy sessions 1, 5, 10, and 15, the primary outcome and putative mediators will be assessed (paper-based or digitally). In addition, the therapists will document changes in concomitant treatments (e.g., medical treatments), SAEs/ESIs that could influence the study therapy, and actions taken (e.g., after consultation with supervisor and/or study head). At the end of treatment (after 20 sessions, regular end of therapy or discontinuation of therapy), participants will complete the post-treatment assessment (paper-based or digitally). Therapists in the blended psychotherapy condition will also complete a brief questionnaire assessing their experience with the implementation of the online platform into treatment. Patients will be invited to a diagnostic telephone interview administered by the study personnel. Six months after the end of treatment, patients will complete a follow-up survey (paper-based or digitally) and will also be invited to another diagnostic interview via telephone. Figure 2 presents the participant timeline and the scheduling of the assessments.

### Sample size {14}

Short-term CBT has demonstrated predominantly small to medium-sized between-group effects on somatic symptom severity in patients with medically unexplained physical symptoms or somatoform disorders (Standardized Mean Difference; *SMD* = 0.37; [18]) and medium- to large-sized within-group effects (*SMD* = 0.80; [83]), as corroborated by a multicenter CBT trial in outpatients with PSS (Cohen’s *d* = 0.70; [51]). Stand-alone internet-delivered CBT yielded small-sized average between-group effects on this outcome (*SMD* = 0.35; [84]) across chronic pain and functional somatic syndromes, whereas evidence from RCTs in individuals with diagnosed/probable SSD demonstrated medium- to large-sized effects (*d* = 0.68 to 0.91) [57, 85]. Evidence from depression research indicates small to medium-sized effects (*d* = 0.32 to 0.51) [34, 86] of added internet interventions to outpatient psychotherapy, which mirrors the treatment setting in the current study. Taken together, we expect the difference between our blended treatment and the enhanced treatment to be *d* = 0.40 for the primary endpoint. Thus, with a power of 80%, and a significance level of 0.05 (two-sided), 200 patients are required, when using a t-test. Because of some variability in effect sizes in the literature and the multicenter study introducing additional heterogeneity we planned the sample size conservatively by not using any correlation between measurements or between outcome and baseline variables. Based on treatment studies in this research field we expect a study drop-out for our primary endpoint (t1) up to 20% [51, 87], resulting in a sample size of *N* = 250 (125 per group). A study in the same setting concluded a similar sample size of 194 [51]. The calculation was performed with SAS 9.4.

### Recruitment {15}

The study centers recruit patients through self-initiated inquiries from individuals seeking psychotherapy in general and through targeted inquiries specifically for the study. To reach potential participants, informational materials (e.g., flyers, posters) are distributed in local medical treatment centers such as medical practices and clinics (e.g., psychosomatic-, pain-, or rehabilitation clinics). The study is also promoted through newspapers, newsletters, and social media. Furthermore, self-help groups are contacted and encouraged to share the informational materials with their members.

## Assignment of interventions: allocation

### Sequence generation {16a}

A randomization list is generated at Interdisciplinary Center for Clinical Trials (IZKS) at the University Medical Center Mainz by means of a validated SAS program (9.4 (SAS Institute, Cary, NC, USA)). The randomization ratio of the treatment group and the control group will be 1:1. Randomization will be stratified by study center. Block randomization with different block size will be applied.

### Concealment mechanism {16b}

Allocation concealment will be ensured through the eCRF-based randomization system. Treatment assignment will be generated automatically only after eligibility has been confirmed and participant enrollment has been completed. The randomization list will be kept in safe and confidential custody at IZKS Mainz and is not accessible to investigators, therapists, assessors, and other site personnel.

### Implementation {16c}

The randomization list will be generated by an otherwise study independent person at the IZKS. Treatment assignment will be performed automatically by the central eCRF-based randomization system. Eligible participants will be enrolled by qualified study personnel at the study sites.

## Assignment of interventions: blinding

### Who will be blinded {17a}

Blinding of patients and therapists is not possible due to the nature of the treatment. The raters (scoring personnel who evaluate the outcome questionnaires) and data analysts will be blinded to treatment allocation. Diagnostic assessments will be conducted by assessors who are independent of treatment delivery but are not formally blinded to treatment allocation. Data analysts will remain blinded until the database has been completed and locked.

### Procedure for unblinding if needed {17b}

No formal unblinding procedure is required because treatment allocation is known to participants and therapists, and blinded data analysts are not involved in clinical decision-making. No circumstances requiring emergency unblinding are anticipated.

## Data collection and management

### Plans for assessment and collection of outcomes {18a}

Surveys will be collected in both paper-pencil and digital form using German versions of the respective scales. After data collection, all data will be entered into the eCRF by the study staff.

#### Primary outcome

The primary outcome, somatic symptom severity, will be measured by the *Screening for Somatoform Disorders* (SOMS-7R [70, 71]), including 54 items (52 items for women, 50 items for men, 50 items for diverse gender identification) assessing the severity of diverse somatic symptoms over the last week on a scale from 0 (not at all) to 4 (very strongly). Additionally, the symptom-related impairment is assessed through three items. An intensity index is calculated as the mean over items 1–54 (range 0–4). In the main validation study with *N* = 325, the previous version SOMS-7 T demonstrated high internal consistency (*α* = 0.92*)* and sensitivity to treatment change [71]. The SOMS-7R has been validated in both clinical and non-clinical samples and is available pre-publication through the main authors, which are part of the study group.

#### Secondary outcomes

Adverse consequences of somatic symptoms will be measured by *Somatic Symptom Disorder-B Criteria Scale* (SSD-12; *α* = 0.95 [73]). The 12 items can be answered from 0 (never) to 4 (very often), resulting in a total sum score from 0 to 48. Somatic symptom coping will be assessed by the coping subscales of the *Pain Coping Questionnaire *(“Fragebogen zur Erfassung der Schmerzverarbeitung”; FESV; *α*_cognitive coping_ = 0.68–0.85, *α*_behavioral coping_ = 0.71–0.81 [75]). Each of the 24 items is scored from 1 (not true at all) to 6 (completely true) yielding a total sum score ranging from 24 to 144. Changes in depression will be assessed by the *Patient Health Questionnaire* (PHQ-9; *α* = 0.89 [74, 88]), which includes 9 items which are scored from 0 (not at all) to 3 (nearly every day) over a 2-week period, resulting in a total sum score from 0 to 27. Changes in anxiety will be measured by the *Generalized Anxiety Disorder* (GAD-7; *α* = 0.92 [76]). The 7 items are scored from 0 (not at all) to 3 (nearly every day) over a 2-week period, and the item scores are summarized to a total score ranged from 0 to 21. Health anxiety will be assessed by the *Whiteley Index* (WI; *α* = 0.80 in a sample of medical outpatients [77, 89]), which consists of 14 items with a dichotomous response format (yes = 1, no = 0). The total sum score ranges from 0 to 14. Pain-related disability will be measured by *Pain Disability Index* (PDI; *α* = 0.86 [44, 90, 91]), modified to assess disability related to PSS not limited to pain. The PDI includes 7 Items which are scored from 0 (no disability) to 10 (maximum disability), resulting in a total sum score ranging from 0 to 70. Changes in symptom related self-efficacy will be assessed by the *Pain Self-Efficacy Questionnaire* (“Fragebogen zur Erfassung der schmerzspezifischen Selbstwirksamkeit”; FESS; *α* = 0.92 [75, 92]), modified to assess self-efficacy related to PSS not limited to pain. The FESS includes 10 items which are scored from 1 (not at all true) to 6 (completely true), resulting in a total score ranging from 10 to 60. Quality of life will be measured using the *World Health Organization Quality of Life -BREF* (WHOQOL-BREF; *α* = 0.72–0.88 across domains [78]), which consists of 26 items scoring from 1 to 5 with varying verbal endpoints (e.g., 1 “not at all”’ to 5 “extremely”) over a 2-week period. Domain scores (physical health, psychological health, social relationship, environment) are calculated and transformed to a 0–100 scale. Changes in experience in social systems will be assessed by the *Experience in Personal Social Systems Questionnaire* (EXIS.pers; *α* = 0.92; [64]),consisting of 12 items which are scored from 1 (not at all) to 6 (entirely) over a 2-week period. Subscale means as well as an overall mean will be calculated.

### Plans to promote participant retention and complete follow-up {18b}

Participants will be informed about the aim and relevance of the study and the importance of completing the treatment and the follow-up survey. This is both for the quality of the study and for their personal chance of benefiting from the study. Furthermore, patients receive 20 EUR as reimbursement for completing the follow-up survey.

### Data management {19}

This trial will be performed using an eCRF. All protocol-required information collected during the trial must be entered by the investigator, or a designated member of the investigating team in the eCRF. All data entries, modifications or deletions will be recorded automatically in an electronic audit trail indicating the individual participant, the original and new values, the reason for and time and date of change, as well as the person executing the change. The system will be secured to prevent unauthorized access to the data or the system. Only people provided with a user ID and a password will be able to enter or change data. Computer hardware and software (for accessing the data) will be maintained at or made available for the site in compliance with applicable regulations. The investigator or a designated member of the investigating team, following review of the data in the eCRF, will confirm the validity of each participant’s data by electronic signature. The architecture of the computer system will be described in the data management plan. During data entry, integrity checks help to minimize entry errors. These data entry checks are based on the data validation plan, signed by the PI. The data entry system allows the trial monitors and data managers to control the entry process with the help of built-in review functions. Comments and requests can be promptly processed by the trial site. Checks for plausibility, consistency and completeness of the data will be performed during data entry. Based on these checks, queries will be produced. Any missing data or inconsistencies will be reported back to the respective site and clarified by the responsible investigator. After completion of data entry and if no further corrections are to be made in the database, the access rights will be withdrawn, and the database will be declared closed and used for statistical analysis. The storage system used during the trial and for archiving (irrespective of the type of media used) should provide for document identification, version history, search, and retrieval. The storage of the electronic data during the trial will be ensured by IZKS Mainz conforming to the legal regulations.

### Confidentiality {27}

The names and health data of the participants are subject to medical professional discretion and the regulations of the applicable laws on data protection. The names of the participants will not be disclosed to the PI. During the clinical trial, participants will be identified solely by means of an individual identification code. The trial site will maintain a personal participant identification list (participant numbers with the corresponding participant names) to enable records to be identified. Trial data (electronic and in paper form) will be handled in strictest confidence. Security procedures will be implemented to prevent disclosure of data to unauthorized people. The appropriate regulations of data protection legislation will be fulfilled in its entirety. The participant will declare in the written consent to release the investigator from the medical professional discretion to enable the attribution of the trial data in case of inspections by health authorities, audits and data monitoring by authorized sponsor representatives.

### Plans for collection, laboratory evaluation and storage of biological specimens for genetic or molecular analysis in this trial/future use {33}

Not applicable as no biological specimens will be collected.

## Statistical methods

### Statistical methods for primary and secondary outcomes {20a}

The *primary endpoint* will be analyzed with a linear mixed-effect model (LMM) including center, use of prescribed psychopharmaceutic (ATC code N06A, yes/no), use of other (non-psychopharmaceutic) medications such as analgesics or antihypertensives (yes/no), mental disorder comorbidity (yes/no), intervention, time point, and the interaction term of intervention and time point as fixed effects, as well as baseline value as a covariate. An autoregressive covariance structure will be used as the primary covariance structure, given the ordered repeated measurements and the expected decrease in correlations over time. Robustness will be assessed using alternative covariance structures. The mixed-effects model uses all available observations and provides valid inference under the missing-at-random assumption. Model assumptions will be checked by residual plots and sensitivity to handling of missing data using multiple imputation. The test used is a linear contrast between the groups at post-treatment (time as categorical variable) and uses a *t*-statistic. Treatment differences will be displayed by adjusted mean differences and 95% confidence intervals. Follow-up data will be analyzed by the LMM approach taking a different contrast. The primary analysis will be performed with the ITT-population. For sensitivity additional subpopulations will be analyzed. Descriptive statistics will also be provided over time by treatment and in total. The primary parameter will also be displayed over time by a boxplot. For sensitivity also response and remission in the SOMS-7R are analyzed. Response will be operationalized as percentage improvement relative to the respective baseline score, (≥ 50% improvement within the pathological range of the SOMS-7R + 25% within the full range [93]). Remission will be coded if patients fulfill response criteria and transition from a dysfunctional to a functional score range in the SOMS-7R. As another remission criterion, we will analyze group differences in diagnostic status (SSD criteria fulfilled) at post-treatment and follow-up. Response and remission will be analyzed within a logistic regression model for each time point with the same covariates as the primary model. Descriptive statistics (absolute and relative frequencies) by time point will also be provided. Continuous secondary outcomes will be analyzed within an analysis model analogous to the primary analysis model and descriptive statistics will be given. Variables expected to have a skewed distribution like working disability days will be analyzed by Wilcoxon Rank Sum test. Categorical outcomes will be analyzed by Χ^2^-tests and will also be displayed by absolute and relative frequencies. Exploratively, health care utilization will be analyzed descriptively at the item level and compared between treatment groups. We will descriptively analyze the extent to which iSOMA is a normal part of the daily working routine of therapists’ post-treatment with the NoMAD. We will test group differences in the type and frequency of self-reported side effects in the NEQ with Χ^2^-tests and independent *t*-test for severity ratings. Potential moderators of the change processes between treatments include (a) demographics, (b) clinical characteristics (e.g., comorbidity, baseline symptom severity, traumatic experience via the CTS), as well as (c) treatment characteristics (e.g., parallel treatments, type of referral, preferences, treatment expectations via GEEE) at baseline. Mediators will be assessed at baseline, during treatment (sessions 1, 5, 10, 15), post-treatment, follow-up. We will examine the temporal, contemporaneous, and between-subject interaction of the therapists’ warmth and competence, treatment expectations, alliance, and psychotherapy outcomes with network models. To test the mechanisms of action of the blended therapy, mediating variables (self-efficacy, disability, and treatment agency) will be analyzed with structural equation modelling (SEM), assuming a lagged effect of a respective mediator on the primary outcome (dual simplex model [94]). This way, indirect effects via different pathways leading to the outcome can be examined. Model fit will be evaluated. Exploratory analyzes will examine further effect-modulating variables (e.g., treatment preferences, patient-, clinical, or treatment characteristics) as moderators (i.e., interaction effects with treatment group) or in the form of subpopulation/sensitivity analyzes (e.g., comorbidity status, treatment length, health care utilization) using the same LMMs as for the main analysis.

### Interim analyzes {21b}

Not applicable since there will be no interim analysis regarding the main objective of the trial.

### Methods for additional analyses (e.g., subgroup analyses) {20b}

Subpopulation analyses will be performed by LMMs similar to the primary analysis model with additional covariates. Subpopulations will be investigated by analyzing the interaction term of the subpopulation and treatment group to optimize treatment selection for individual patients. The determination of the Minimal Clinically Important Difference for the SOMS-7R will be done precision based (using the Standard Error of Measurement), distribution based (0.5 Standard Deviation), and anchor based [95, 96]. To test robustness of effects, we will examine LMMs for the following sample: patient per protocol: regularly completed therapy and primary outcome completed at baseline, end of therapy, and 6-month follow-up.

### Methods in analysis to handle protocol non-adherence and any statistical methods to handle missing data {20c}

The primary analysis model is able to deal with missing at random data. Multiple imputation will be used as a sensitivity analysis. One hundred imputed datasets will be generated using fully conditional specification (chained equations). The imputation model will include variables from the primary analysis model as well as baseline, clinical, and outcome-related variables considered relevant for predicting missingness and/or outcome values. Each imputed dataset will be analyzed using the primary analysis model, and results will be combined according to Rubin’s rules. Further details regarding the imputation model will be prespecified in the statistical analysis plan.

### Plans to give access to the full protocol, participant level-data and statistical code {31c}

Anonymized data may be made available for scientific purposes upon justified request, after completion of all publication processes and under a contractual data reuse agreement (Access Class 3 “secure data” according to the guidelines by the German Psychological Association, DGPs [97]). Metadata and anonymized, decontextualized cumulative (sub)data are intended to be made publicly accessible in a repository (e.g., ZPID, PsychArchives), including a contact address for inquiries from other researchers and information on the terms of reuse.

## Oversight and monitoring

### Composition of the coordinating center and trial steering committee {5d}

This multicenter study is coordinated by the “Clinical Psychology, Psychotherapy and Experimental Psychopathology” working group at Johannes Gutenberg University Mainz. The trial steering committee (TSC) is composed of the PI and the appointed co-applicants. The PI has overall responsibility for all study-related activities, including the training of the study therapists and nominates adequately qualified members of the investigating team and must instruct and supervise them in order to ensure that they are adequately informed about relevant information regarding the trial, especially the trial protocol and the investigators brochure. Each participating center appoints a study coordinator or coordination team responsible for the implementation of study procedures, recruitment of participants and data management. To ensure effective communication and organization, all centers participate in a monthly online meeting to discuss current developments, clarify logistical issues and address open points. Data management will be overseen by the IZKS at the University Medical Center Mainz. Clinical monitoring, including on-site visits, will also be conducted by the IZKS.

### Composition of the data monitoring committee, its role and reporting structure {21a}

The independent Data Safety Monitoring Board (DSMB) will include a psychologist and eHealth expert, a specialist in psychosomatic medicine, and a biostatistician. The DSMB will be regularly provided with all safety aspects of the trial and will review the safety data. Twice a year a meeting or a telephone conference of the board will be scheduled to review the progress of the trial, to ensure adherence to the protocol, and to advise whether to continue, modify, or stop the trial. Protocol adherence will be systematically assessed during clinical supervision by the evaluation of treatment videos. In particular, the DSMB will assess whether or not the recruitment plan is on target. Based on relevant information from within the trial or from other sources the DSMB may stop the trial at any time. The DSMB will provide independent recommendations to the PI and the TSC, who will be responsible for communicating relevant information and progress reports to the funding organization.

### Adverse event reporting and harms {22}

Psychotherapy is generally considered safe, even though discussing personal issues can sometimes evoke strong or unpleasant emotions. The researchers do not anticipate any excess risks or negative effects for participants from the treatment or study procedures. Participants will receive high-quality outpatient psychotherapy and complete established, standardized questionnaires. There will be no special physical demands on study participation (no blood or saliva sampling, no medication or placebo administration, no invasive measurements). Potential side effects of the treatment will be systematically assessed throughout the trial by therapists using a checklist at the time of occurrence and during scheduled visits (sessions 1, 5, 10, and 15) as well as at the post-assessment and the follow-up assessment (administered by study personnel). This is done to monitor any SAEs or events that may negatively influence the course of treatment. The following SAEs will be assessed systematically: (1) death, (2) attempted suicide, (3) intentional self-injury, (4) intoxication with a psychotropic substance requiring medical treatment, (5) life-threatening event, (6) unplanned hospitalization, (7) persistent or significant disability or incapacity. In addition to SAEs, certain ESIs will also be recorded, even if they do not meet any of the above-mentioned criteria for seriousness. These include the following: suicidal ideation or plans, the development of an acute psychosis, and the breakdown of a close or important relationship. Therapists are instructed to discuss all these events and potential subsequent actions in supervision. Additionally, patient-rated potential side effects of the treatment will be assessed via standardized self-report (NEQ) at post-assessment.

### Frequency and plans for auditing trial conduct {23}

Quality management will be conducted by the IZKS Mainz during the study continuously as a risk-based mixture of remote and on-site monitoring to ensure protocol adherence, patients’ safety, and integrity of the data. To initiate the trial, the monitor will visit all participating trial sites. The monitor shall assure that the investigators and members of the investigating staff understand all requirements of the protocol and their legal responsibilities. Each trial site will be visited by the monitor at regular intervals to check compliance with the trial protocol, Good Clinical Practice and legal aspects. The presence of informed consents will be checked for every trial participant. The monitor will perform a risk-based review the entries into the eCRF for completeness and correctness and verify the entries by cross-checking against the source documents. Source data review and source data verification can only be conducted if the technical prerequisites are given and a valid regulatory approval exists. At the remote monitoring visits, primarily administrative aspects and plausibility could be checked. Source data review and source data verification can only be conducted if the technical prerequisites are given and a valid regulatory approval exists.

### Plans for communicating important protocol amendments to relevant parties (e.g., trial participants, ethical committees) {25}

All substantial modifications that could affect the implementation of the study, study materials, or participant safety will be submitted as formal amendments to the ethical committees of the leading center as well as to those of the participating recruitment centers. If approved, these changes will be reported in the trial register and in the trial paper. Any amendments or changes will be communicated to all parties involved in the study. If modifications directly impact participants, the informed consent documents will be revised accordingly, and all participants will be clearly informed.

### Dissemination plans {31a}

The results of the study will be communicated to relevant expert societies such as the EURONET-SOMA, the DGPs and the State Chambers of Psychotherapists. Results will be presented at national and international conferences to both medical and psychological experts to enhance awareness of effective (blended) treatments. We expect interest in the result from health insurance companies. The results will be reported according to the CONSORT criteria. In addition, the results will be published as part of the final report to the German Research Foundation (Deutsche Forschungsgemeinschaft, DFG) and will be accessible to all those interested on their pages. Finally, further data may be shared upon reasonable request and after all publication processes of our results will be completed.

## Discussion

The proposed trial aims to close a significant gap in the treatment of SSD by investigating the efficacy of a new blended psychotherapy. It is among the first trials to investigate outpatient psychotherapy for this diagnosis, which is still not implemented in the German health care system. This approach integrates a modular internet-based intervention into outpatient CBT and will be one of the first to systematically assess a manualized CBT approach for SSD. Thus, the trial directly addresses the limited availability of effective treatment options for this highly prevalent disorder [98]. The multicenter implementation across outpatient treatment centers throughout Germany ensures broad service coverage, thereby strengthening the transferability of the results to clinical practice. Compared to “passive” bibliotherapy, the add-on internet intervention will provide a range of interactive and tailored therapeutic content, multimedia features (e.g., videos, interactive exercises) as well as persuasive design features (e.g., progress bar, skill collection as achievements, lesson reminders). Together, these features of rather “active” self-help aim to promote patient engagement, encourage the integration of adaptive health behaviors into daily life, and enhance self-efficacy in coping with symptoms, which are key mechanisms of therapeutic change [41, 50]. Evidence indicates a dose-response relationship for psychotherapy of SSD [37], which is why additional online therapeutic elements could intensify and condense psychotherapy [99] without proportionally increasing therapeutic resources and costs in the same way as additional on-site sessions, making it a “smart augmentation.” Besides these add-ons, both study arms share a significant treatment component, potentially challenging expected treatment effects. Furthermore, practical challenges may arise, such as the semi-flexible integration of the internet-based intervention into manualized therapy protocols, which requires therapeutic effort in terms of planning, guidance, and monitoring. Also, while comorbid disorders are largely permitted, they will not explicitly be targeted within the scope of the intensive short-term therapy. However, the focus on SSD and the short-term nature of the treatment can foster implementability of the treatment regime.

The identification of prescriptive factors (i.e., moderators/mediators) will help therapists to identify patients who rather benefit from more standard or blended treatment forms. Given that patients with SSD rarely access traditional psychotherapy [22], due to both structural and disorder-specific barriers, low-threshold self-help elements delivered with an innovative medium may enhance access to and acceptability of psychotherapy [100] and give patients greater treatment options. Regarding treatment preferences and acceptance, the combination of face-to-face and digital components is expected to address a more clinically representative patient sample [101] than in former trials using stand-alone internet interventions (e.g., [84]) and may help overcome one of the key barriers to digital interventions – the absence of personal contact [102]. Moreover, the blended format could also be associated with greater acceptance by therapists [26].

However, the trial may also face some methodological and clinical challenges. First, the SSD population is inherently heterogeneous, encompassing a wide and diverse range of somatic symptoms [103] and comorbid psychological conditions [6, 12, 13]. Second, maintaining patients’ treatment adherence over the medium-duration treatment period may be challenging, particularly among individuals with low digital affinity or fluctuating motivation. To address this, the trial implements supportive elements such as reminders, therapist encouragement, and user-centered module design, reflecting persuasive design principles that have been shown to enhance adherence to web-based interventions [104]. Third, while blended interventions are promising in theory, their successful implementation depends on therapist training, digital infrastructure, and organizational support factors [105, 106] that will need to be addressed in parallel to clinical evaluation.

While conducted as a randomized controlled trial with standardized treatment protocols, our trial applies somewhat broader inclusion criteria and allows concomitant treatments such as medication, thus increasing external validity and enhancing generalizability to routine care—at the same time requiring methodological consideration as outlined in our study design. For future implementation, iSOMA must be accessible via secure platforms and supported by training. The included therapist training may serve as a foundation for broader dissemination. In this context, the trial will help to further harness the potential of digitally supported health care by equipping a larger number of therapists across Germany with the skills to integrate high-quality digital tools into psychotherapy. In summary, the trial offers the opportunity to advance both clinical care and implementation science by evaluating an integrated blended psychotherapy for SSD. Its findings may inform broader dissemination strategies for digital interventions and contribute to more accessible and effective treatment options for underserved SSD patient populations.

### Trial status

The recruitment phase has started in November 2025, and it is planned to continue until August 2027. The end of the study is currently scheduled for January 2029.

## Data Availability

As the data involve sensitive (clinical routine) information, anonymized data will be made available for scientific purposes upon justified request, following the completion of all publication processes and subject to a contractual data use agreement. This corresponds to access class 3, “secure data”, as defined by the DGPs guidelines [97]. Metadata (i.e., a description of the dataset) and anonymized, decontextualized, cumulative (partial) data will be made publicly accessible via a repository (e.g., ZPID, PsychArchives), including a contact address for researcher inquiries and information on data use conditions.

## Abbreviations

SSD: Somatic Symptom Disorder
CBT: Cognitive behavioral therapy
SOMS-7R: Screening for Somatoform Disorders
DRKS: German Clinical Trials Register
DFG: German Research Foundation
PSS: Persistent somatic symptoms
SAE: Serious adverse event
ESI: Event of Special Interest
PHQ-15: Patient Health Questionnaire, somatic symptom version
PDI: Pain Disability Index
PI: Principal investigator
MATD: Method of assessing treatment delivery
EURONET-SOMA: European Research Network to Improve Diagnosis, Treatment and Health Care for Patients with Persistent Somatic Symptoms
ITT: Intention-to-treat
SSD-12: Somatic Symptom Disorder – B Criteria Scale
FESV: Pain Coping Questionnaire
FESS: Pain Self-Efficacy Questionnaire
PHQ-9: Patient Health Questionnaire – depression scale
GAD-7: Generalized Anxiety Disorder
WI: Whitely Index
WHOQOL-BREF: World Health Organization Quality of Life questionnaire
HCU-Q: Health Care Utilization Questionnaire
EXIS.pers: Experience in Personal Social Systems Questionnaire
TAI: Therapeutic Agency Inventory
CTS: Childhood Trauma Screener
GEEE: Generic Rating Scale for Previous Treatment Experiences, Treatment Expectations, and Treatment Effects
W&C: Warmth & Competence Scale
WAI-SR: Working-Alliance Inventory – short revised
ZUF-8: Client Satisfaction Questionnaire
NoMAD: Normalization Measure Development Questionnaire
NEQ: Negative Effects Questionnaire
DIPS: Diagnostic Interview for Psychological Disorders
Mini-DIPS: Short form of the Diagnostic Interview for Psychological Disorders
SMD: Standardized Mean Difference
IZKS: Interdisciplinary Center for Clinical Trials
eCRF: Electronic Case Report Form
LMM: Linear Mixed-Effect Model
SEM: Structural Equation Modelling
SD: Standard Deviation
DGP: German Psychological Association
TSC: Trial Steering Committee
DSMB: Data Safety Monitoring Board
